# Implant-Related Complications Using Uniaxial Implants In Pediatric Spinal Deformity Surgery

**DOI:** 10.7759/cureus.16715

**Published:** 2021-07-29

**Authors:** Masayoshi Machida, Brett Rocos, David E Lebel, Jeremie Nallet, Reinhard Zeller

**Affiliations:** 1 Department of Orthopaedic Surgery, Hospital for Sick Children, Toronto, CAN

**Keywords:** adolescent idiopathic scoliosis (ais), pediatric spine, complication of treatment, paediatrics orthopaedic surgeon, implant breakage

## Abstract

Introduction: The successful surgical treatment of paediatric spinal deformity relies on robust anchors to achieve correction. Uniaxial pedicle screws are designed with articulation between the screw head and screw shaft, thus reducing the risk of anchor failure whilst permitting corrective manoeuvres. The purpose of this study was to describe the incidence, nature, and chronology of implant-related complications in pediatric spinal deformity treated with uniaxial pedicle screws.

Methods: A retrospective radiographic analysis was carried out on paediatric patients treated for spinal deformity with more than two years of follow-up. Each was treated with posterior instrumented spinal fusion (PISF) using a uniaxial pedicle screw system by a single surgeon at a single institution. Surgical records, post-operative radiographs, and follow-up documentation were scrutinised for details of the implants used, implant failure, and revision procedures.

Results: Three hundred and eighty-nine eligible patients with a mean follow-up of 3.3 years were identified. The mean anchor density was 1.7. Seven implant complications were observed. Early complications (<12 months) occurred in four cases and late (>12 months) in three cases. None of the early complications were associated with non-union. Two early and two late complications required revision surgery to manage implant failure and non-union. Patients who underwent fusion across the lumbosacral junction showed a higher than expected rate of implant-related complication (P=0.02).

Conclusion: This study shows that there is a rate of implant-related failure of 1.8% after PISF with uniaxial implants in pediatric spinal deformities. There is a distinction between early and late implant-related complications, with early failure being due to loss of construct integrity, whereas late failure is due to pseudarthrosis and construct fatigue.

## Introduction

The surgical treatment of paediatric spinal deformity continues to evolve as the understanding of the precise shape and biomechanics of the deformity improves and implants become more versatile [1-3]. Nonetheless, deformity surgery remains a challenging proposition and implant-related complications are not uncommon [4-6].

The most fragile part of the spine-implant construct is the anchor points to the vertebrae. Anchor design has evolved to minimise the risk of failure, beginning with wires, progressing through hooks, and culminating in pedicle screws [4,7]. Early pedicle screw designs were fixed angles, whereby there was no articulation between the screw head and the shaft. These offered a superior correction of vertebral rotation when compared to hooks, however, had the potential for pedicle fracture during corrective manoeuvres. The consequent failure of the anchor led to loss of the correction and revision surgery in many cases [8].

The polyaxial screw was created to resolve these drawbacks. These devices are designed with articulation between the screw head and screw shaft, permitting the screw head to approach the rod (in addition to bringing the rod to the screw), facilitating rod seating, and reducing the strain on the screw-bone interface [8,9]. Unfortunately, the disadvantage of these screws is their comparatively inferior ability to control vertebral rotation during correction [8].

A compromise between fixed angle and polyaxial designs is the uniplanar screw. This design accommodates variations of the pedicle screws relative to the rod only in the sagittal plane. This degree of freedom allows for adjustment in the sagittal plane thus preventing the destruction of the bone-implant interface through screw toggling [7,10].

The implant system used in this study (SpineVision PLUS [SpineVision, Antony, France]) takes advantage of this technology and is one that is unique in the design of its rod-screw interface. The screw head is shaped with a convex saddle at the bottom of the implant in common with other systems, however, rather than a set screw being used to compress the rod into the head, the rod is compressed into the screw with a handle then an inverted U-shaped clip (the ‘clip’) is placed and seated under a lip on either side of the head. This configuration permits the rod to slide in a cranial-caudal direction with up to 20° of sagittal rotation during any correction manoeuvres. Once these movements are complete, a grub screw in the centre of the clip is tightened to prevent any additional cranial-caudal migration of the rod and secure the correction. The constructs are then reinforced with crosslinks as required.

To date, no reports describe an investigation of implant-related complications for such a system. The purpose of this study was to describe the incidence, nature, and chronology of implant-related complications in paediatric spinal deformity treated with uniaxial pedicle screws.

## Materials and methods

Local research ethics board approval was obtained. A retrospective radiographic analysis of the implants used, timing, and nature of any implant failures (implant loosening, fracture, pullout or rod-anchor interface breakage) in paediatric patients treated for spinal deformity were identified using a local hospital database which was carried out by a fellowship-trained spinal orthopaedic surgeon. Other complications were also recorded. Each patient was treated with a posterior instrumented spinal fusion (PSIF) using a uniaxial pedicle screw system (SpineVision PLUS [SpineVision, Antony, France]) by a single surgeon at a single institution with additional hooks used when pedicle morphology prevented screw placement. Inclusion criteria comprised age less than 18 years at the time of PSIF with a minimum of two years completed follow-up. Demographics, body weight (BW), body mass index (BMI), American Society of Anaesthesiologists (ASA) grade, activities of daily living (ADL), and medical histories were recorded. Surgical records, post-operative radiographs, and follow-up documentation were scrutinised for details of any revision procedures.

The data were analysed using STATA (v14.0, StataCorp, College Station, TX). The Mann-Whitney U test and Fisher’s exact tests were used to analyse numerical and categorical data. P-values of less than 0.05 were considered to indicate a statistically significant difference.

## Results

Three hundred and eighty-nine eligible patients with a mean follow-up of 3.3 years (range 2-9.4 years) were identified, each undergoing instrumentation with the SpineVision PLUS (SpineVision, Antony, France) system. Of the 389, 75.5% were idiopathic, 4.6% congenital, 5.6% neuromuscular, and 14.1% syndromic deformities (Table 1). The mean age at surgery was 14.0 years (95%CI 13.7-14.2). Sixty-eight percent of patients were Risser grade 4 or higher and 7.7% showed open triradiate cartilage. Over 95% of patients were able to independently stand preoperatively.

**Table 1:** Characteristics of the cohort. The characteristics of the cohort by diagnosis.

	Total (n=389)	Idiopathic (n=293)	Neuromuscular (n=26)	Syndromic (n=49)	Congenital (n=21)
Age (years)	14.0 (13.7–14.2)	14.4 (14.2–14.6)	13.9 (13.2–14.7)	13.4 (12.6–14.2)	9.1 (7.0–11.2)
Gender					
Male	65	36	7	17	5
Female	324	257	19	32	16
Risser sign					
0	61	24	7	16	14
1	19	14	2	3	0
2	26	24	0	2	0
3	34	30	1	2	1
4	117	101	6	8	2
5	131	99	10	18	4
Follow-up (years; 95%CI)	3.3 (3.1–3.4)	3.1 (3.0–3.3)	3.5 (2.9–4.0)	3.4 (3.0–3.9)	4.1 (3.3–5.0)
Previous laminectomy	1	0	1	0	0
Prior anterior release	19	8	1	4	6
Preoperative halo traction	11	2	0	9	0
Mean anchor density per vertebra (95%CI)	1.73 (1.70–1.75)	1.8 (1.8–1.8)	1.5 (1.4–1.7)	1.5 (1.4–1.5)	1.5 (1.4–1.7)
Lumbosacral fusion (n)	13	0	6	4	3

Eight percent of the cohort had undergone previous spinal surgery, none with the same instrumentation system. Corrective surgery utilised the SpineVision PLUS system with an average of two rods, 15 uniaxial pedicle screws, six hooks, and two crosslinks used in each construct (Table 1). The mean anchor density was 1.7 per vertebral level (range 0.7-2.2). This represented 5808 pedicle screws, 2158 hooks, 802 crosslinks, and 810 rods. Thirteen patients (3.3%) were fused across the lumbosacral junction, of which the distal-most anchor was an iliac screw in five. Iliac crest and local bone graft were used in 87.5% of cases, with additional tibial autograft in the remaining 12.5%.

Seven implant complications were observed, comprising three screws (0.07%), two hooks (0.09%), one crosslink fracture (0.12%), and one-rod breakage (0.13%). This represents 1.8% of cases; three patients had an idiopathic deformity, two congenital deformities, and two neuromuscular deformities (Table 2).

**Table 2:** Implant-related complications. The frequency of implant-related complications.

Complications	Total	Early	Late
Rod breakage	1	0	1
Pedicle screw pullout	1	0	1
Iliac screw pullout Iliac screw breakage	2	2	0
Hook slip	2	2	0
Crosslink fracture	1	0	1
Total (n=389)	7 (1.8%)	4 (1.8%)	3 (0.8%)
Idiopathic (n=293)	3 (1.0%)	1 (0.3%)	2 (0.7%)
Congenital (n=21)	2 (10.0%)	2 (9.5%)	0
Neuromuscular (n=26)	2 (7.7%)	1 (3.8%)	1 (3.8%)

**Figure 1:**
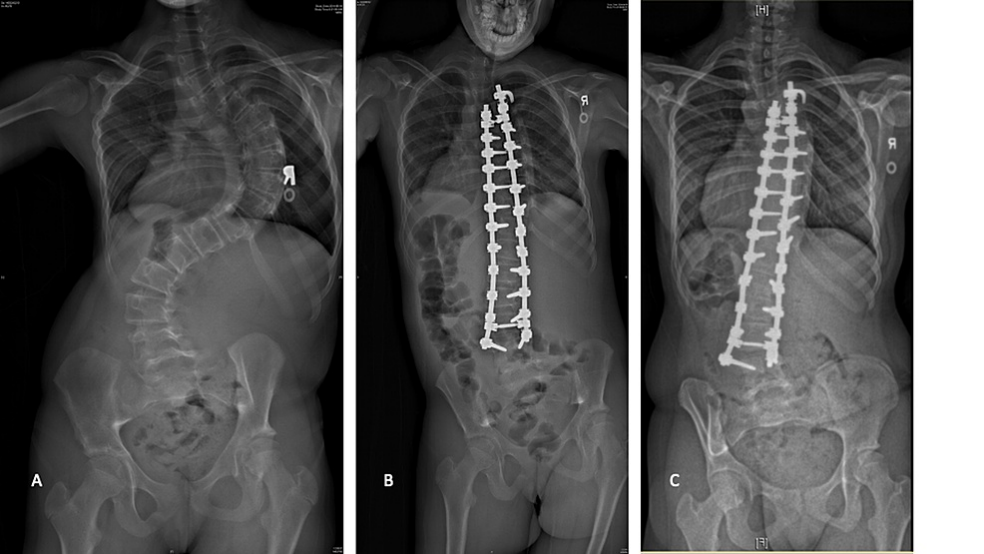
The patient diagnosed with adolescent idiopathic scoliosis showing pullout of the distal pedicle screws associated with non-union. The patient was diagnosed with adolescent idiopathic scoliosis showing pullout of the distal pedicle screws associated with non-union. (A) Pre-operative AP radiograph; (B) immediate post-operative AP radiograph; (C) AP radiograph showing pullout of the left L4 pedicle screw.

**Figure 2:**
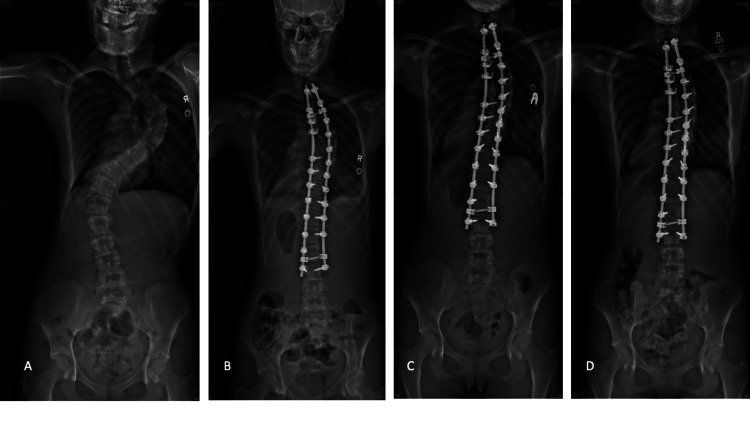
Patient diagnosed with adolescent idiopathic scoliosis with post-operative stable hook pullout. A patient diagnosed with adolescent idiopathic scoliosis with post-operative stable hook pullout: (A) pre-operative AP radiograph; (B) immediate post-operative AP radiograph; (C) AP radiograph showing a slipped proximal hook at the right T2; (D) no change in the position of the spine at two years following surgery.

Within the seven complications, there was one case of lumbar pedicle screw pullout with an associated nonunion (Figure 1A-1C); one iliac screw breakage after a patient returned to activity earlier than instructed (return to activity protocol were no sporting activity at all for one year, followed by low-impact non-competitive activities for the second post-operative year); one iliac screw pullout in a patient with cerebral palsy; a single incident of rod breakage associated with non-union in a syndromic scoliosis; two incidents of hook slip without the loss of correction (Figure 2A-2D); and one crosslink fracture after a patient was treated for infantile scoliosis without clinical sequelae.

Categorising implant failures into early (<one postoperative year) and late (>one post-operative year), early complications occurred in four cases and late in three cases. The broken iliac screw, two slipped hooks and iliac screw pullout all occurred within 12 months of surgery, and the broken rod, broken crosslink, and pedicle screw pullout all occurred more than 12 months following surgery. The late broken rod and pedicle screw pullout were associated with non-union. None of the early complications were associated with non-union, and there were no cases of postoperative clip detachment.

Two early complications required revision surgery (iliac screw pullout, broken iliac screw), and two late complications required revision surgery to manage implant failure and non-union (rod breakage, pedicle screw pullout). Patients who underwent fusion across the lumbosacral junction showed a higher than expected implant-related complication rate (P=0.02).

## Discussion

In this series of 389 cases of paediatric spinal deformity treated with the SpineVision PLUS system, we observed an implant-related complication in 1.8% of cases or seven out of 9,578 implants (0.07%). Of these, three were late complications associated with pseudarthrosis. The remainder were early complications associated with failure of the implant mechanisms or interfaces. Patients fused to the ilium were at a greater relative risk of encountering implant-related complications than those in whom the lowest instrumented level was more proximal (relative risk 11.6, 95%CI 2.5- 54.2). Four of the seven implant failures required revision surgery, two for non-union in addition to the revision of the failed implants.

Previous reports that have included patients treated with a range of alternative implant systems have found that the rate of implant-related complications after PSIF for spinal deformity surgery in paediatric patients is rare, ranging from 0.6% to 1.1% in idiopathic cases, 1.5% in congenital scoliosis and 2.1-12.5% in neuromuscular deformity, though they are likely underreporting of these due to the methodology employed [5,11,12]. In general, these complications are described as screw pull-out or screw or rod breakage related to non-union.

No previous reports have detailed the failure of the implant mechanisms as a cause for failure. There is an important distinction to be made between early (<one year) and late (>one year) complications, a distinction made because 12 months is a reasonable time for fusion to occur in the lumbar spine [13]. Early complications were those identified on plain radiographs and indicated failure of the implant system without loss of correction or a need for revision surgery, a similar definition to that used by O’Neill et al. in the cervical spine [14]. In contrast, late complications were failures of the construct due to non-union and generally required revision to restore mechanical stability and facilitate fusion.

These data confirm that there is a higher risk of implant-related complications when spinal fusions are extended across the lumbosacral junction. Moreover, the rate of complication of the distal-most anchors is lower than that seen in previous studies, which have shown that the rate of loosening of the iliac screw in neuromuscular spine deformity surgery is between 1.8% and 39.2% and the incidence of iliac screw breakage between 21.4% and 23.6% [15,16].

There are limitations in this study in addition to its retrospective design. This study investigates a single implant system with a unique rod-anchor interface, used by a single surgeon, which may introduce a bias to the conclusions. Furthermore, although the system is uniaxial, it has a unique rod-screw interface that is not directly comparable to other systems. Lastly, one of the early failures was due to a patient failing to follow post-operative instructions which may influence conclusions. Further investigations would benefit from including alternative surgical implant systems with different rod-screw interface mechanisms, used by multiple surgeons to increase the generalisability of the conclusions.

## Conclusions

This study shows that there is a rate of implant-related failure of 1.8% after PISF with uniaxial implants in paediatric spinal deformities. It identifies that there is a distinction between early and late implant-related complications, with early failure being due to loss of construct integrity, whereas late failure is due to pseudarthrosis and construct fatigue. Similar studies should be undertaken with other implant systems to evaluate more precisely their respective mechanical characteristics.
